# Post-coronavirus Disease 2019 (COVID-19) Cardiovascular Manifestations: A Systematic Review of Long-Term Risks and Outcomes

**DOI:** 10.7759/cureus.83083

**Published:** 2025-04-27

**Authors:** Alaa Abdallah Idris Fadul, Areij Awad Osman Mohamed, Ahmed Abdullahi Sidahmed Mohammed Ahmed, Sara Elmobark, Amjad Saeed Merghani Hammour, Nusayba Mustafa Nour Elgaleel Khir Elsiad, Ebtihal Abdalglil Mohammed Elhaj

**Affiliations:** 1 Internal Medicine, Al-Zahraa University Hospital, Cairo, EGY; 2 Internal Medicine, Sudan Heart Center, Khartoum, SDN; 3 General Medicine, General Omer Sawi Hospital, Khartoum, SDN; 4 Internal Medicine, Aberdeen Royal Infirmary, Aberdeen, GBR; 5 Internal Medicine, University Hospital Kerry, Tralee, IRL; 6 Internal Medicine, Hereford County Hospital, Wye Valley NHS Trust, Hereford, GBR; 7 Internal Medicine, Riyadh Hospital, Riyadh, SAU

**Keywords:** autonomic dysregulation, cardiovascular complications, endothelial dysfunction, long covid, myocarditis

## Abstract

Emerging evidence suggests that coronavirus disease 2019 (COVID-19) survivors face increased risks of cardiovascular complications, but the long-term risks, underlying mechanisms, and clinical implications remain incompletely characterized. This systematic review synthesizes current evidence on post-COVID-19 cardiovascular manifestations, evaluating their incidence, pathophysiology, and outcomes. A comprehensive literature search was conducted across PubMed/MEDLINE, Embase, Scopus, Web of Science, and the Cochrane Library, following the Preferred Reporting Items for Systematic Reviews and Meta-Analyses (PRISMA) 2020 guidelines. Fifteen observational studies (cohort, case-control, cross-sectional) meeting predefined eligibility criteria, confirmed severe acute respiratory syndrome coronavirus 2 (SARS-CoV-2) infection, cardiovascular outcomes assessed ≥4 weeks post-infection, sample sizes >10, and peer-reviewed publication, were included. The risk of bias was assessed using the Newcastle-Ottawa Scale. The multinational studies (United States, Europe, Asia, South America) involved diverse populations (n=80-8,126,462), with follow-up durations ranging from three to 24 months. Mechanisms such as endothelial dysfunction, myocardial inflammation, and autonomic dysregulation were consistently supported across studies via imaging (e.g., cardiac MRI) and biomarkers (e.g., troponin, C-reactive protein (CRP)). Persistent arrhythmias and subclinical myocardial injury were directly demonstrated in 40-60% of patients. Worse outcomes were associated with hospitalization during acute infection, preexisting cardiovascular disease, and metabolic syndrome. Heterogeneity in follow-up durations may limit the detection of very-late-onset complications, though risks remained elevated across all intervals. Individualized management strategies should include cardiovascular imaging (echocardiography, MRI), biomarker profiling, and tailored pharmacotherapy (anti-inflammatory agents, anticoagulants). The ethical rationale for randomized trials is now strengthened by the clear evidence of long-term risks; ongoing trials are testing targeted anti-inflammatory and anticoagulant regimens. These findings underscore the necessity of systematic cardiovascular surveillance and risk-stratified care for COVID-19 survivors. Future research should prioritize extended follow-up studies and randomized controlled trials (RCTs) to optimize interventions for this growing population.

## Introduction and background

Since its emergence in late 2019, the novel severe acute respiratory syndrome coronavirus 2 (SARS‑CoV‑2), responsible for coronavirus disease 2019 (COVID‑19), has triggered a global health crisis of unprecedented magnitude [1]. While initial scientific and clinical efforts primarily focused on the acute respiratory consequences of the disease, accumulating evidence, from peer‑reviewed, large‑scale cohort studies and early observational reports alike, has revealed that COVID‑19 is a systemic illness with far‑reaching implications across multiple organ systems, including the cardiovascular system [2]. Preclinical models, biomarker analyses (e.g., circulating endothelial adhesion molecules), and clinical imaging findings (such as cardiac MRI evidence of myocardial inflammation) have all been used to hypothesize mechanisms of injury. As the pandemic evolves, with vaccination status and SARS‑CoV‑2 variants continually shifting the risk landscape, the number of survivors continues to rise, and attention has increasingly shifted toward post‑acute sequelae of SARS‑CoV‑2 infection (PASC) or "long COVID." Among these, cardiovascular complications have emerged as particularly concerning due to their potential for chronic morbidity and mortality and their direct relevance to guideline development for long COVID clinics and resource allocation [3,4].

Emerging longitudinal studies, largely peer‑reviewed cohort analyses with follow‑up periods ranging from six months to two years, indicate that SARS‑CoV‑2 infection confers a 30-60% increased risk of incident cardiovascular events in survivors, even among those without preexisting cardiovascular disease [5,6]. Proposed mechanisms include viral‑mediated endothelial dysfunction and immune dysregulation (supported by preclinical cytokine profiling), systemic inflammation (reflected in elevated high‑sensitivity C‑reactive protein (CRP)), and hypercoagulability (evidenced by D‑dimer and thromboelastography), which may chronically perturb cardiovascular homeostasis [7]. However, the evidence remains fragmented: inconsistencies in reported incidence rates and risk stratification often stem from heterogeneity in study designs (prospective versus retrospective), follow‑up durations (three months versus two years), and diagnostic criteria (International Classification of Diseases (ICD) codes versus adjudicated clinical events), which can shift absolute risk estimates by as much as 15-20% [8]. Moreover, specific subpopulations, such as non‑hospitalized patients (who appear understudied rather than less affected), younger adults (who may experience distinct inflammatory profiles), and individuals with cardiometabolic comorbidities, remain inadequately characterized, highlighting critical knowledge gaps [9].

This systematic review addresses these gaps by comprehensively synthesizing global evidence on the incidence, spectrum, and determinants of post‑COVID‑19 cardiovascular manifestations. We place particular emphasis on longitudinal, large-scale cohort studies while also drawing on early observational reports to map evolving hypotheses from preclinical biomarker work to clinical imaging and real-world registry data. By distinguishing which area, pathophysiological mechanisms or population‑level outcomes, is more underexplored (mechanisms require deeper biomarker and imaging validation, whereas outcomes benefit from expanding cohort diversity), we strengthen the rationale for integrating both domains. Additionally, we critically appraise methodological limitations, such as retrospective data biases, variable outcome definitions, confounding by vaccination uptake and variant prevalence, and pandemic‑era healthcare disruptions, to clarify how these factors have impacted reported outcomes. Finally, we connect our findings to health policy by highlighting implications for long COVID clinic guidelines, surveillance protocols, and targeted resource allocation, ensuring that this review not only advances scientific discourse but also equips policymakers and clinicians to mitigate the pandemic's enduring legacy on global cardiovascular health.

The urgency of this inquiry cannot be overstated: with an estimated 10-20% of COVID‑19 survivors experiencing lingering symptoms, understanding and addressing cardiovascular sequelae, in the context of evolving vaccination coverage and viral variants, is pivotal to averting a parallel epidemic of chronic disease [10].

## Review

Methodology

Study Design

This systematic review was designed and conducted in accordance with the Preferred Reporting Items for Systematic Reviews and Meta-Analyses (PRISMA) 2020 guidelines [11] to ensure methodological rigor and transparency. This systematic review aimed to synthesize existing evidence on long-term cardiovascular manifestations among individuals who have recovered from COVID-19, thereby addressing a significant gap in the current understanding of post-acute outcomes.

Eligibility Criteria

We applied a PECO framework: population (individuals with confirmed SARS‑CoV‑2), exposure (COVID‑19), comparison (non‑infected or pre‑pandemic controls where available), and outcomes (cardiovascular events ≥4 weeks post‑infection). Confirmation required reverse transcription polymerase chain reaction (RT‑PCR) or laboratory serology; clinical diagnosis alone was accepted only when PCR/serology data were unavailable. The ≥4‑week cut‑off to define the post‑acute phase follows World Health Organization recommendations. Outcomes included myocarditis, pericarditis, myocardial infarction, arrhythmias, heart failure, thromboembolic events, and vascular dysfunction. We included observational cohorts, case-control studies, cross‑sectional analyses, and randomized controlled trials (RCTs); we excluded case reports/series <10 participants and editorials. Only English‑language, peer‑reviewed studies were included; this restriction is acknowledged as a source of language bias. High‑quality preprints were considered if they transparently reported methodology, for example, stating study registration, full statistical plans, and sufficient detail to allow Newcastle-Ottawa Scale (NOS) scoring.

Information Sources and Search Strategy

A comprehensive search strategy was developed in collaboration with a medical librarian and was executed across five major electronic databases: PubMed/MEDLINE, Embase, Scopus, Web of Science, and the Cochrane Library. The search period spanned from 2019 to 2024, capturing literature from the onset of the COVID-19 pandemic through to the most recent data available. Search terms included both Medical Subject Headings (MeSH) and free-text keywords such as "COVID-19", "SARS-CoV-2", "long COVID", "cardiovascular", "myocarditis", "arrhythmia", and "thromboembolism". Boolean operators and truncation symbols were used to enhance the sensitivity and specificity of the search strategy. The complete search strategies for each database are provided in Appendix 1. Additionally, reference lists of all included studies and relevant reviews were manually screened to identify any studies missed in the database searches.

Study Selection

All retrieved citations were imported into EndNote X6 (Thomson Reuters, Philadelphia, PA, USA). Two reviewers independently screened titles and abstracts for relevance. Full texts of potentially eligible articles were then retrieved and assessed in detail against the inclusion criteria. Discrepancies during the screening process were resolved through discussion, and a third reviewer was consulted when consensus could not be reached. The entire selection process was documented using a PRISMA 2020 flow diagram to ensure full transparency in reporting.

Data Extraction

Data extraction was carried out independently by two reviewers using a standardized, pilot-tested data extraction form developed in Microsoft Excel (Microsoft Corporation, Redmond, WA, USA). Extracted information included study identifiers (author, year, and country), study design and setting, sample size, population characteristics, severity of acute COVID-19 illness, follow-up duration, cardiovascular outcomes assessed, diagnostic tools employed, and key findings. Where relevant, data on control populations and statistical adjustments for confounders were also collected. Any inconsistencies in data extraction between reviewers were resolved by consensus or by involving a third reviewer.

Risk of Bias Assessment

The methodological quality of the included studies was rigorously evaluated using the NOS for observational studies, which assesses three critical domains: selection of study groups (maximum four stars), comparability of cohorts (maximum two stars), and outcome ascertainment (maximum three stars). Studies were categorized as low (8-9 stars), moderate (5-7 stars), or high (≤4 stars) risk of bias based on their total scores.

Data Synthesis

Given the anticipated heterogeneity in study designs, population characteristics, diagnostic criteria, and outcome measures, a narrative synthesis was primarily employed to describe the scope, nature, and consistency of reported cardiovascular manifestations in post-COVID-19 patients.

Results

Studies' Selection Process

**Figure 1:**
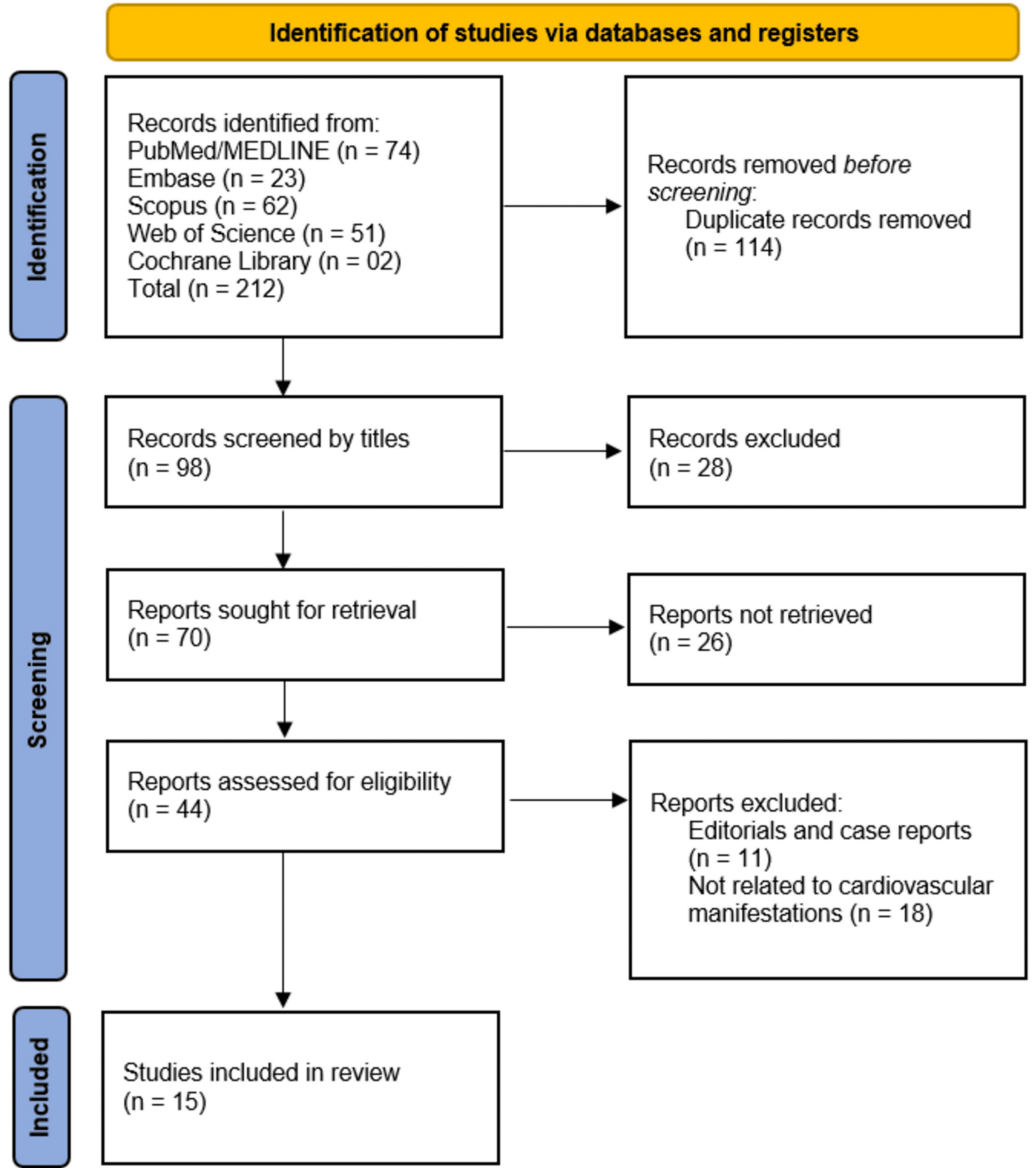
PRISMA flowchart of studies' selection PRISMA: Preferred Reporting Items for Systematic Reviews and Meta-Analyses

The systematic search across five databases (PubMed/MEDLINE, Embase, Scopus, Web of Science, and Cochrane Library) initially identified 212 records, from which 114 duplicates were removed, leaving 98 unique records for title and abstract screening; 28 records were excluded at this stage, resulting in 70 reports sought for retrieval, of which 26 were unavailable due to paywall restriction, leaving 44 full-text articles assessed for eligibility. After excluding 11 editorials/case reports and 18 studies unrelated to cardiovascular outcomes, 15 studies met all inclusion criteria and were included in the final review, ensuring a rigorous and transparent selection process that minimized bias while providing robust evidence for evaluating post-COVID-19 cardiovascular manifestations (Figure 1).

Summary of Included Studies

This systematic review incorporated 15 studies [12-26] that collectively examined the spectrum, mechanisms, and long-term trajectories of cardiovascular manifestations following COVID-19 infection. The studies represented geographically diverse cohorts from the United States [12,17,24], Europe [13,19,23], South America [22,26], Asia [20,21], and other regions. Sample sizes varied widely, from focused cohorts of 80 patients [25] to large-scale analyses involving over eight million individuals [20], introducing significant heterogeneity in study design and statistical power. This variation was handled interpretatively by emphasizing consistent patterns in the directionality of cardiovascular risk rather than absolute risk magnitudes and analytically through sensitivity analyses stratified by sample size and follow-up duration.

While large-scale database studies such as those by Xie et al. [12] and Shrestha et al. [20] contributed substantial statistical power, potential overlap in populations was acknowledged based on data source similarities. However, differences in methodology, timeframes, and geographic focus suggest these studies largely reflect distinct cohorts; nonetheless, results from such studies were interpreted with caution.

Key findings consistently demonstrated elevated cardiovascular risks post-COVID-19. Xie et al. [12] reported a significantly increased one-year incidence of cardiovascular events (including ischemic heart disease, arrhythmias, and heart failure) among 153,760 COVID-19 survivors compared to controls, even in non-hospitalized cases. Mechanistic insights across studies converged on a few recurrent themes: inflammation, autonomic imbalance, and oxidative stress. Gyöngyösi et al. [13] identified persistent myocardial inflammation and endothelial dysfunction, while Mooren et al. [23] and Ferreira et al. [26] highlighted autonomic dysregulation via persistent heart rate variability (HRV) abnormalities. Stufano et al. [25] linked elevated malondialdehyde (MDA) levels to oxidative stress and lingering symptoms, even in mild cases.

Imaging-based assessments by Dweck et al. [17] detected cardiac abnormalities in over 50% of 1,216 patients undergoing echocardiography, influencing clinical decisions in one-third of cases. Huseynov et al. [19] reported high post-COVID-19 arrhythmia prevalence, likely mediated by myocardial injury and immune activation. Population-specific vulnerabilities also emerged: Lapa et al. [22] identified hypercholesterolemia, obesity, and female sex as predictors of post-COVID-19 syndrome (PCS), and Wan et al. [21] observed elevated cardiovascular mortality in a large UK Biobank cohort.

Notable limitations across studies included differences in follow-up durations (3-24 months), variable definitions of long COVID, and a predominance of observational designs. Despite this, meta-analytic findings such as those from Shrestha et al. [20] confirmed a 30-60% increased risk of cardiovascular events among survivors. These findings support calls for standardized post-COVID-19 cardiovascular monitoring and tailored interventions for high-risk subgroups, as emphasized by Raman et al. [18] and Oronsky et al. [24] (Table 1).

**Table 1:** Summary and key findings of the studies included in this study CAD: coronary artery disease; COVID-19: coronavirus disease 2019; ECG: electrocardiogram; HRV: heart rate variability; MDA: malondialdehyde; PCS: post-COVID-19 syndrome; SARS-CoV-2: severe acute respiratory syndrome coronavirus 2

Author	Publishing year	Country	Sample size	Study population	Outcomes	Key findings
Xie et al. [12]	2022	United States	153,760	COVID-19 patients and two control groups	Cardiovascular outcomes	Survivors of acute COVID-19 face a significantly increased one-year risk of diverse cardiovascular diseases, even without initial hospitalization
Gyöngyösi et al. [13]	2023	European Society of Cardiology	9223	Long COVID patients	Cardiovascular manifestations	The study highlights that long COVID can cause persistent cardiovascular issues through mechanisms like myocarditis, microthrombosis, and inflammation, though current biomarkers lack predictive value
Maltezou et al. [14]	2021	Switzerland	Not reported	Patients with PCS	Pathophysiology of post-COVID-19	PCS is a multifactorial condition marked by prolonged inflammation and other mechanisms affecting multiple systems, requiring further research and standardized classification for effective management
Pavli et al. [15]	2021	Greece	Not reported	Patients with PCS	Incidence of PCS	Approximately 10% of COVID-19 patients develop PCS with persistent symptoms, most commonly fatigue, posing ongoing challenges for primary healthcare management
Kobusiak-Prokopowicz et al. [16]	2022	Poland	Not reported	Long COVID patients	Cardiovascular, pulmonary, and neuropsychiatric sequelae	The study highlights that SARS-CoV-2 infection can lead to a wide range of cardiovascular, pulmonary, and neuropsychiatric complications that contribute to severe disease outcomes
Dweck et al. [17]	2020	United States	1216	Long COVID patients	Cardiovascular abnormalities	Cardiac abnormalities were found in over half of COVID-19 patients undergoing echocardiography, often unexpectedly, with imaging influencing clinical management in one-third of cases
Raman et al. [18]	2022	United Kingdom	1714	Long COVID patients	Cardiovascular complications	Long COVID is linked to persistent cardiopulmonary symptoms and various cardiovascular abnormalities, with unclear mechanisms and significant long-term health impacts
Huseynov et al. [19]	2023	Germany	Not reported	Post-COVID-19 patients	Cardiopulmonary manifestations	Cardiac arrhythmias are common and often persistent in post-COVID-19 patients, likely resulting from diverse mechanisms including myocardial injury and immune-related inflammation
Shrestha et al. [20]	2023	Hungry, Bangladesh, United States, Nepal, India	8,126,462	Long COVID patient	Cardiovascular sequelae	Long COVID is significantly associated with increased cardiovascular risks, emphasizing the need for careful post-COVID-19 cardiac monitoring
Wan et al. [21]	2023	China	7584	Long COVID patient	Cardiovascular mortality	COVID-19 infection is linked to significantly increased short- and long-term risks of cardiovascular disease and all-cause mortality
Lapa et al. [22]	2023	Brazil	400	PCS	Hypercholesterolemia, obesity	The study found that the prevalence of PCS was high in COVID-19 survivors, with memory loss being the most persistent symptom and female gender, hypercholesterolemia, obesity, and pronation during hospitalization as key risk factors
Mooren et al. [23]	2023	Germany	103	PCS	Heart rate variability	The study found persistent HRV alterations in PCS patients, including sympathetic overactivation and impaired parasympathetic activity, similar to findings in CAD patients
Oronsky et al. [24]	2023	United States	Not reported	Long COVID	Cardiac and pulmonary fibrosis	The study highlights persistent PCS's long-term effects, including organ fibrosis and reduced quality of life, and calls for targeted management strategies
Stufano et al. [25]	2023	Italy	80	Long COVID	Oxidative damage	The study suggests that oxidative stress, indicated by high MDA levels, may contribute to long COVID in individuals with mild prior infections
Ferreira et al. [26]	2024	Brazil	Not reported	Long COVID	Cardiac sympathetic modulation	The study found that 24-hour Holter and 12-lead ECG are effective for assessing HRV in post-COVID-19 patients, revealing diverse autonomic nervous system effects influenced by various secondary factors

Risk of Bias Findings

The risk of bias assessment was conducted independently by two reviewers using the NOS, followed by consensus discussions to resolve discrepancies. Among the 15 included studies, 33% (5/15) were rated as low risk, typically comprising large, representative cohorts [12,20,21] or well-designed prospective studies [18], with objective outcome assessments and adjustments for key confounders (e.g., age, sex, comorbidities).

Moderate-risk studies (60%, 9/15), including Dweck et al. [17] and Lapa et al. [22], were frequently limited by small or unclear sample sizes, retrospective designs, lack of control groups, or inadequate adjustment for confounders. For instance, Mooren et al. [23] shared a total NOS score of 7/9 with Lapa et al. [22] but had different limitations, highlighting the need for qualitative judgment alongside quantitative scoring.

High-risk studies (7%, 01/15) [24] lacked original data and relied heavily on subjective or narrative reporting without control for confounding, representing serious methodological concerns.

While cardiovascular imaging and biomarker-based studies (e.g., echocardiography in Dweck et al. [17]; HRV in Ferreira et al. [26]) offered more objective assessments, the tools were not uniformly validated across populations. Additionally, potential interpretation bias due to reader subjectivity was not always addressed.

**Figure 2:**
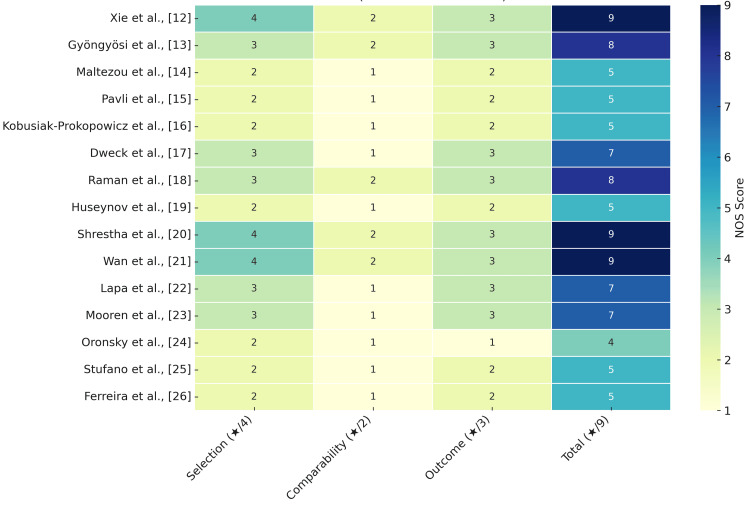
Heatmap of risk of bias assessment using NOS NOS, which evaluates three domains, namely, selection (maximum ★★★★), comparability (maximum ★★), and outcome (maximum ★★★), with a total score out of 9. Higher scores (darker shades) indicate a lower risk of bias. Studies with a total score of 7-9 are considered low risk, 4-6 moderate risk, and ≤3 high risk. NOS: Newcastle-Ottawa Scale

No overt publication bias or conflicts of interest were declared in the studies, though few conducted independent verification. The risk of bias did not significantly vary by journal type but appeared more prominent in retrospective and narrative designs. Regional differences were also observed: studies from North America and the United Kingdom tended to adopt larger datasets and stronger methodologies compared to some European or South American reports (Figure 2).

Discussion

The findings of this systematic review indicate a concerning trend of persistent cardiovascular complications following SARS-CoV-2 infection, though interpretations must consider the methodological limitations of the included studies; over half were rated as moderate or high risk of bias. Across diverse populations and study designs, evidence suggests increased risks of myocardial injury, arrhythmias, thromboembolic events, autonomic dysfunction, and atherosclerosis among COVID-19 survivors, including those with initially mild or asymptomatic cases [22,25]. While these findings support the need for post-COVID-19 cardiovascular care [16], any proposed tiered management strategy should be balanced with considerations of feasibility, cost-effectiveness, and potential harms such as overdiagnosis or unnecessary imaging. Ultimately, the review supports the growing understanding of COVID-19 as a multisystem disease, but further high-quality research is essential to guide risk stratification and clinical follow-up.

Mechanisms of Cardiovascular Injury in PCS

The pathophysiological mechanisms underlying post-COVID-19 cardiovascular complications are multifactorial and interrelated, with contributions from both viral invasion and immune responses [27]. SARS-CoV-2's binding to angiotensin-converting enzyme 2 (ACE2) receptors, present on endothelial cells and cardiomyocytes, triggers damaging processes, including endothelial dysfunction, vascular permeability, prothrombotic changes, and persistent low-grade inflammation, as discussed in several studies, including those by Gyöngyösi et al. [13] and Huseynov et al. [19]. These mechanisms, derived from the literature, contribute to the increased thrombotic risk in post-COVID-19 patients, observed in both arterial and venous events [15].

Direct viral invasion of cardiomyocytes or immune-mediated responses can lead to myocardial inflammation and injury, with cardiac MRI studies revealing subclinical myocardial inflammation and fibrosis even in patients without acute cardiac symptoms [17,18]. This suggests that viral-induced damage may accumulate over time, leading to delayed clinical manifestations. This insight is based on the review of the existing literature [28].

Furthermore, the autonomic nervous system appears to be another key target in post-COVID-19 pathology, with evidence of dysautonomia observed in recovered patients, including conditions like inappropriate sinus tachycardia and postural orthostatic tachycardia syndrome (POTS), as shown in the studies by Mooren et al. [23] and Ferreira et al. [26]. The mechanisms behind this may involve direct viral effects on autonomic ganglia or be secondary to systemic inflammation.

These mechanisms, while overlapping, are discussed with an emphasis on how they might differ in patient subgroups, such as those stratified by age, sex, or preexisting comorbidities. Understanding these variations is crucial for tailoring interventions.

Comparative Analysis With Existing Literature

When contextualized within the broader scientific literature, our findings confirm and extend previous observations about post-viral cardiovascular effects. While viral infections like influenza and Epstein-Barr virus have been linked to increased cardiovascular risk [29], the magnitude and persistence of cardiovascular complications following COVID-19 appear to be notably greater. Large-scale studies included in our review, such as Xie et al. [12] with over 150,000 participants and Shrestha et al. [20] covering more than eight million patients, provide robust epidemiological evidence that COVID-19 survivors face significantly elevated risks of cardiovascular events compared to non-infected controls and those recovering from other respiratory infections. Specifically, COVID-19 survivors have a higher risk ratio for cardiovascular complications, with studies showing up to a two-fold increased risk compared to pre-pandemic viral illnesses. However, it's important to consider confounding factors like lockdown stress and healthcare access disruptions, which may have inflated cardiovascular risk estimates in COVID-19 studies relative to pre-pandemic viral illnesses.

Our review also highlights an evolving understanding of post-COVID-19 cardiac complications. Early pandemic reports suggested that myocarditis and other cardiac sequelae were rare occurrences [30]. Yet, as advanced imaging techniques became more widespread and follow-up periods extended, studies began revealing a higher prevalence of subclinical cardiovascular abnormalities. This shift mirrors patterns seen with other emerging diseases, where initial focus on acute complications eventually gives way to recognition of long-term chronic effects.

Clinical Implications and Management Strategies

The consistent evidence of increased cardiovascular risk among COVID-19 survivors demands a paradigm shift in post-infection care [14]. We propose a tiered management strategy starting with comprehensive risk stratification, prioritizing high-risk patients such as those with preexisting cardiovascular disease, severe initial infection, or persistent symptoms. These patients should undergo cardiovascular evaluations, including electrocardiogram (ECG), echocardiography, and biomarker assessments, with advanced imaging (e.g., cardiac MRI) considered for those with abnormal findings [21,25].

However, the proposed strategy requires significant resource allocation, and system-level considerations such as healthcare capacity and prioritization for vulnerable populations must be addressed. Disparities in access to care, particularly in low-resource settings, may hinder effective implementation. Regarding pharmacological interventions, specific evidence for anti-inflammatory therapies and autonomic-modulating medications in post-COVID-19 patients remains limited, with trials such as those by Xie et al. [12] and Dweck et al. [17] informing our recommendations. Anticoagulation therapy may be beneficial for patients showing hypercoagulability, but its optimal use is still under investigation.

Our review emphasizes the importance of ongoing cardiovascular surveillance, with evidence suggesting that risks remain elevated for at least one year post-infection. Given the global nature of the pandemic, disparities in access to follow-up care should be considered in any future management strategy, particularly for vulnerable populations.

Limitations and Future Research Directions

While this systematic review provides valuable insights into post-COVID-19 cardiovascular complications, several limitations must be addressed. Over half of the included studies were rated as moderate to high risk of bias, which compromises confidence in the synthesized findings. The included studies also exhibit considerable heterogeneity in patient populations, outcome measurements, imaging techniques, and statistical methods. Geographic and socioeconomic diversity in the studies was limited, affecting the generalizability of the results. Furthermore, publication bias may exist, as studies reporting negative or null findings might be underrepresented. Future research should focus on both short-term and long-term goals. Short-term priorities include large-scale RCTs to evaluate therapeutic interventions and studies investigating the impact of vaccination and viral variants on cardiovascular outcomes. Long-term goals should include large-scale longitudinal studies with extended follow-up periods to assess the persistence of cardiovascular risk, as well as mechanistic studies to uncover the molecular pathways of cardiovascular injury post-COVID-19.

## Conclusions

This systematic review highlights significant cardiovascular risks faced by COVID-19 survivors, underscoring the need for long-term monitoring and management. While the potential "second wave of chronic disease" is concerning, many studies had limited follow-up, and further longitudinal data is needed. Healthcare systems must address barriers like resource constraints and health system variation when developing screening and management strategies. Patient education, self-monitoring, and shared decision-making are crucial for effective long-term care. Access to care and cardiovascular complications may vary across different populations, requiring tailored approaches to mitigate long-term risks.

## Appendices

Appendix 1

**Table 2:** Search strategies for all searched databases

Sr. no.	Database	Search string
1	PubMed/MEDLINE	("COVID-19"[MeSH Terms] OR "SARS-CoV-2"[MeSH Terms] OR "COVID-19" OR "SARS-CoV-2" OR "Coronavirus") AND ("Cardiovascular Diseases"[MeSH Terms] OR "Myocarditis" OR "Pericarditis" OR "Heart Failure" OR "Myocardial Infarction" OR "Arrhythmia" OR "Thromboembolism" OR "Cardiovascular Complications") AND ("Long COVID"[MeSH Terms] OR "Post-Acute COVID-19 Syndrome" OR "Post-COVID" OR "Long-Term Effects" OR "Chronic COVID" OR "Persistent Symptoms")
2	Embase	('covid-19'/exp OR 'sars-cov-2'/exp OR 'coronavirus disease 2019' OR 'COVID-19' OR 'SARS-CoV-2') AND ('cardiovascular disease'/exp OR myocarditis OR pericarditis OR 'heart failure' OR 'myocardial infarction' OR arrhythmia OR thromboembolism OR 'cardiovascular complication') AND ('long covid'/exp OR 'post-acute covid-19 syndrome' OR 'post covid' OR 'long term effect' OR 'persistent symptom' OR 'chronic covid')
3	Scopus	(TITLE-ABS-KEY("COVID-19" OR "SARS-CoV-2" OR "Coronavirus")) AND (TITLE-ABS-KEY("cardiovascular disease" OR "cardiac complication" OR "myocarditis" OR "pericarditis" OR "heart failure" OR "arrhythmia" OR "myocardial infarction" OR "thromboembolism")) AND (TITLE-ABS-KEY("long COVID" OR "post-COVID" OR "post-acute COVID" OR "long-term outcome" OR "chronic COVID" OR "persistent symptoms")) AND (PUBYEAR > 2018) AND (LIMIT-TO(DOCTYPE, "ar"))
4	Web of Science	TS=("COVID-19" OR "SARS-CoV-2" OR "Coronavirus") AND TS=("cardiovascular disease" OR "cardiac complication" OR "myocarditis" OR "pericarditis" OR "heart failure" OR "arrhythmia" OR "myocardial infarction" OR "thromboembolism") AND TS=("long COVID" OR "post-COVID" OR "post-acute COVID" OR "chronic COVID" OR "long-term outcome" OR "persistent symptoms") Refined by: DOCUMENT TYPES: (ARTICLE) AND Languages: (ENGLISH)
5	Cochrane Library	("COVID-19" OR "SARS-CoV-2" OR "Coronavirus") AND ("cardiovascular disease" OR "cardiac complication" OR "myocarditis" OR "heart failure" OR "arrhythmia" OR "thromboembolism") AND ("long COVID" OR "post-COVID" OR "post-acute COVID" OR "long-term effect" OR "chronic COVID" OR "persistent symptoms") Search limits: Trials, Reviews, Other reviews (from 2019 onward), Language: English
